# Cognitive Abilities and Their Implications for Language and Speech Disorders—Insights From Phoniatric Practice

**DOI:** 10.1002/pdi3.70072

**Published:** 2026-09-23

**Authors:** Ghada Tarek Orabi, Raghda Abdelsalam Nagaty Hanady

**Affiliations:** ^1^ Department of Research on Children with Special Needs, Medical Research and Clinical Studies Institute National Research Centre Cairo Egypt; ^2^ Clinical Pathology Department Research Institute of Ophthalmology Giza Egypt

**Keywords:** cognitive abilities, delayed language development, language and speech disorders, nonverbal IQ, specific language impairment, stuttering, verbal IQ, working memory

## Abstract

Children with language and speech disorders account for approximately 5%–12% of children have language and speech disorders globally. Understanding the cognitive abilities of these children is crucial for identifying how cognitive skills influence speech fluency, language production, and planning effective intervention programs. Subtle differences in cognitive performance have been noted to affect developmental language skills. Therefore, we aimed to compare the cognitive abilities of children with language impairments and stuttering to typically developing controls. Our study was conducted on 69 children. The children were divided into three groups. The first and second groups consisted of children with language/speech impairments and fluency disorders, whereas the third group consisted of typically developing peers. We used standardized methods to assess the children's speech, language, fluency, and cognitive skills. The results demonstrated that although children with language impairments and stuttering had IQ scores that fell within the normal range, their verbal IQ and working memory scores showed specific differences from their typically developing peers. These findings suggest that cognitive abilities should be assessed and monitored in children with communicative disorders, even when overall IQ scores fall within the average range. Subtle differences in cognitive skills may affect speech fluency and language development.

## Graphical abstract

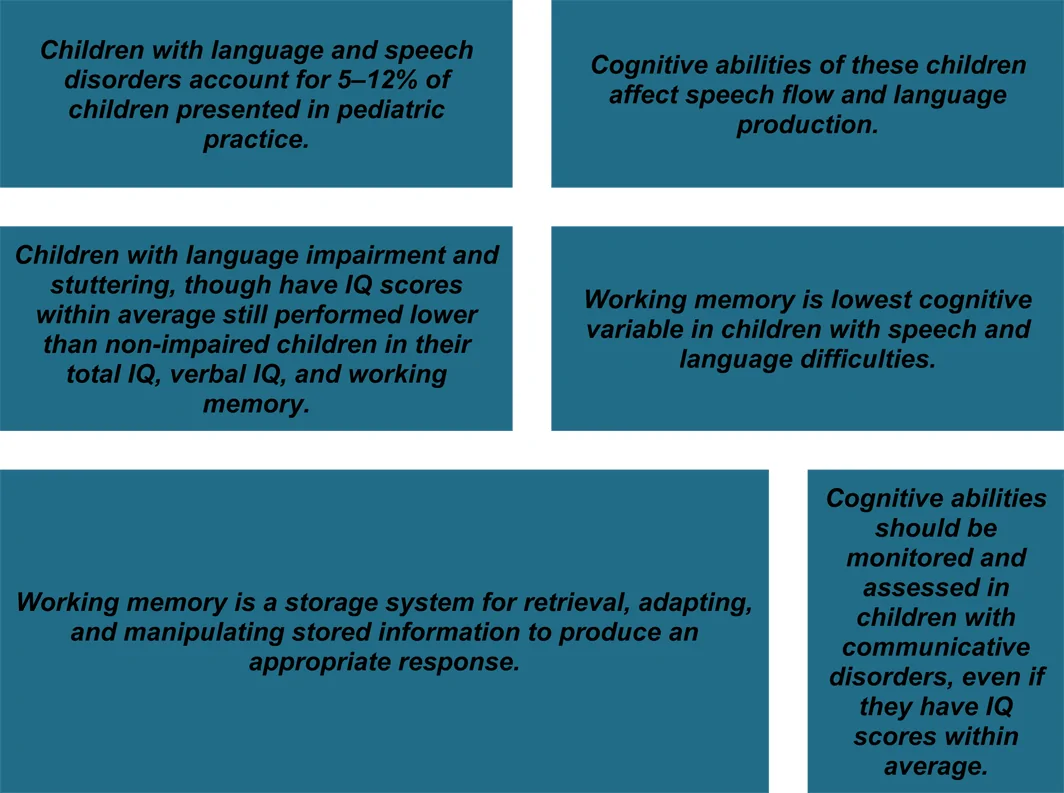

## 1 Introduction

Children with different communication disorders constitute approximately 5%–12% of general pediatric referrals [1]. About 10.8% children between the ages of 3 and 6 years are estimated to have language and speech disorders [2]. The 2016 National Health Survey [3] reports that 8% of children aged 3 to 17 experience communication problems related to either language development or speech fluency, with males being more frequently affected than females. Early identification of language and speech disorders is essential to plan and design appropriate intervention services. Pediatricians are often the first healthcare providers from whom parents and caregivers seek advice to initiate referral to specialized services [4]. In Egypt, estimates of the prevalence of communication disorders (including language delay and speech dysfluency) among pediatric patients are approximately 10% [5]. Two of the most common clinical representations in communicative disorders are specific language impairment (SLI)—also known as delayed language disorder—and speech fluency problems such as stuttering [6]. Durget and Tekin reported that nearly 80% of children presented with communicative disorders had isolated developmental language problems (i.e., language delay without other neurological deficits, nor with global cognitive delay) [6]. Specific language impairment is defined as delayed language development in the absence of any other neurological, neurodevelopmental, or cognitive causes [7] such as autism, cognitive delay, or other neurological causes, e.g., cerebral palsy [8]. SLI affects approximately 7.6% of kindergarten‐aged children, who otherwise demonstrate typical nonverbal cognitive abilities [7]. SLI has been linked to learning disabilities such as reading and writing difficulties known as dyslexia and dysgraphia [9]. Phonological and lexical deficits in SLI are due to slower phonological processing, poor phonological memory skills, and difficulties in learning phoneme–grapheme associations, which lead to reading difficulties [10]. Children with SLI typically display poor semantics, with syntactic errors resulting in short sentence structures with multiple phonological errors leading to reduced speech intelligibility, and/or pragmatic deficits that affect functional use of language [11]. On the other hand, stuttering is not considered a developmental language delay but rather a disruption of speech fluency [12]. Although children who stutter have typical linguistic development, their disrupted speech flow affects their psychological well‐being and communicative ability, resulting in reduced expressive language performance than peers without stuttering [13]. Children with SLI or stuttering often present with typical cognitive development, and their IQ scores generally fall within the average range [14, 15]. However, research suggests subtle differences in their cognitive and executive functioning compared to their typically developing peers [16, 17]. Children with language impairment show lower performance in working memory tasks [18], verbal working memory, and phonological processing [19]. It has been hypothesized that slower processing of verbal input is due to alterations in procedural memory systems [20], leading to impairments in grammar, semantics, and phonological accuracy, and consequently, the speech becomes unintelligible (i.e., strangers or unfamiliar listeners do not fully understand their verbal output) [18]. Similarly, children who stutter demonstrated lower performance in tasks involving working memory, cognitive flexibility, and verbal IQ compared to fluent peers [21, 22]. Working memory limitations may hinder their ability to control speech flow and fluency, while simultaneously performing other cognitive tasks. Identifying cognitive and executive deficits in children with language and speech problems is important in understanding the underlying mechanisms of language development and speech control. This knowledge also helps in developing effective rehabilitation programs that address these cognitive weaknesses alongside conventional speech and language therapy. The subtle cognitive deficits in children with communication disorders, are frequently overlooked in treatment planning [23]. A comprehensive understanding of specific cognitive and executive differences in communication and language disorders is necessary to tailor therapy approaches accordingly. Previous studies often assessed cognitive abilities using either verbal or nonverbal measures separately [24]. The present study aimed to provide a more comprehensive assessment of cognitive functions using the Stanford–Binet Intelligence Scales, which include subscales assessing problem‐solving, reasoning, knowledge, and working memory in both verbal and nonverbal domains [25], This study aimed to compare the differences in cognitive abilities between children with language impairments, children with stuttering, and typically developing controls, in order to delineate any subtle differences in their underlying cognitive skills.

## 2 Methods

### 2.1 Study Participants

This cross‐sectional case‐control study was conducted in 2023–2024. Children were recruited from the Phoniatrics Clinic, Center of Medical Excellence, National Research Centre, Cairo, Egypt. We included children with developmental stuttering or developmental phonological speech disorder as per clinical examination, results of the stuttering severity index, and speech intelligibility tests. Children with neurodevelopmental disorders (e.g., autism spectrum disorder, attention deficit hyperactivity disorder [ADHD]), children with intellectual disability or chronic medical conditions affecting intellectual function (such as chronic diseases, thyroid dysfunctions, and chronic anemia), and children with motor speech deficits (such as dysarthria), were all excluded. The control group was recruited from surrounding schools: they were typically developing children with typical phonological and language development, without any reported history of developmental disabilities, and with no signs of developmental stuttering as per examination, and reports from their parents, and teachers. They were matched with cases in terms of age and sex. The participants underwent medical examinations to exclude children with any associated neurological conditions, and to rule out any chronic medical conditions that could influence attention or cognitive functioning (e.g., anemia, or thyroid dysfunction). The subjects from the three groups had their history, including their place of residences, parents' jobs, and school enrollment and attendance, collected. All the children chosen for the study came from similar urban areas in western Cairo, Egypt, as this was the area where the research facility clinic was located. Their places of residence had continuous water supply, electricity, and Internet access. All the children in the current study were enrolled in schools and the Phoniatrics Clinic and attended regularly. They all had at least one parent with a stable job as income. To ensure the matched controls were similar in their socioeconomic backgrounds, the controls were chosen from schools surrounding the research facility, and from neighborhoods close to the subjects. The 3 group of children were mostly similar in socio‐economic backgrounds as per their places of residence, schools, and stable income households. Written informed consent was obtained from all caregivers prior to participation. The study protocol was approved by the Medical Research Ethics Committee, National Research Center, Cairo, Egypt (Federal Wide Assurance No. FWA00014747; RH DIR: 2,017,103,002; Final Decision: 01,451,223).

### 2.2 Laboratory Investigations

All children underwent a panel of laboratory tests to exclude potential metabolic or nutritional factors that might affect cognitive performance. These included a complete blood count (CBC) to rule out anemia; serum iron and ferritin to assess iron status; serum vitamin B12 and folate as indicators of neurocognitive support; thyroid function tests (TSH, free T4, free T3) to rule out hypo‐ or hyperthyroidism that could impair neuromotor coordination; and serum vitamin D, zinc, and magnesium levels to assess micronutrient status.

### 2.3 Language and Speech Assessments

All children's language abilities were evaluated through a series of standardized assessments, including spontaneous speech evaluation to assess spontaneous speech, attention, and responsiveness to the examiner; receptive language assessment to evaluate understanding of different semantic and syntactic categories via picture identification tasks; and expressive language assessment to evaluating semantic knowledge, sentence production, and word repetition via picture naming and description.

For children with specific language impairments and multiple phonological errors, formal testing of speech intelligibility was conducted using the Arabic Speech Intelligibility Test for Children [26] to assess how clearly and accurately a child could produce words and sentences to unfamiliar listeners. The speech intelligibility test was developed to assess language and phonological development in the Egyptian Arabic dialect. The child was required to name 50 pictures from three different sets. The examiner transcribed the child's responses verbatim, what was understood from his utterances rather than what the child intended, without the examiner seeing the exact picture presented to the child (although the examiner had prior knowledge word sets in each group). Separate scores of each of the three sets (one including words with frontal sounds, second with backward sounds, and third set of sentences), as well as total scores of all utterances, were calculated as percentages. The percentage reflected the proportion of intelligible speech out of total utterances. A child would be considered below age‐appropriate speech intelligibility developmental milestones if the speech intelligibility was below 50% at 3 years, below 75% at 4 years, below 85% at 5 years, and below 100% at 6 years and older. These thresholds indicated excessive phonological deficits associated with language impairment, whereas percentages at or above these levels indicated age‐appropriate thresholds of speech intelligibility. A higher percentage of intelligible speech in the children's expressive language was indicative of typical developmental thresholds. Children with stuttering and disrupted speech fluency were evaluated using the Arabic Stuttering Severity Instrument (ASSI) [27]. Scores ranged from 0 to 45, with the following severity classifications: ≤ 22, mild stuttering; 23–30, moderate stuttering; and ≥ 31, severe stuttering. Children in the control group were required to have normal fluency of speech with no disruptions in speech flow, with scores ranging from 0‐10 in the stuttering severity index scale, indicating zero to few sound repetitions, or disruptions during their speech.

### 2.4 Cognitive Assessment

All participants' cognitive abilities were assessed using the Arabic adaptation of the Stanford–Binet Intelligence Scales, Fifth Edition (SB5) [28]. The SB5 provides a comprehensive verbal and non verbal assessment of the major factors that compromise cognitive abilities. This IQ test provides results on eight subtests of fluid reasoning, knowledge, quantitative reasoning, visual‐spatial reasoning, working memory, verbal IQ, non‐verbal IQ and full scale IQ.

### 2.5 Statistical Methods

Data management, analysis, and graph generation were conducted using RStudio Software (version 4.3.2). Numerical data were summarized as means ± standard deviations (SD) or medians and interquartile ranges (IQR), as appropriate. Categorical data were expressed as frequencies and percentages. Normality of numerical variables was assessed using the Kolmogorov–Smirnov and Shapiro–Wilk tests. For categorical data, comparisons among independent groups were performed using the Chi‐square test or Fisher's exact test, as appropriate. For normally distributed numeric variables, group comparisons were conducted using Student's *t*‐test (two groups) or one‐way ANOVA (more than two groups). For non‐normally distributed numeric variables, the Mann–Whitney *U* test (two groups) or Kruskal–Wallis H test (more than two groups) was applied. When the Kruskal–Wallis test showed significance, Dunn's post hoc test with Bonferroni correction was used for multiple pairwise comparisons. A *p*‐value < 0.05 was considered statistically significant.

## 3 Results

### 3.1 Study Population

The study included 69 children, equally divided into three groups: Group 1, Children with stuttering (*n* = 23; 33.3%), Group 2, Children with poor speech intelligibility (*n* = 23; 33.3%), and Group 3, Typically developing controls (*n* = 23; 33.3%) with no significant differences among groups regarding age or sex distribution (Table 1). Table 1 shows that the three groups—Stuttering, Poor Speech, and Control—are comparable in age and sex, with no significant differences (*p* = 0.374 and *p* = 0.814, respectively).

**TABLE 1:** Demographic and clinical characteristics of the children across the three groups.

Variables	Group of stuttering (*n* = 23)	Group of poor speech (*n* = 23)	Control roup (*n* = 23)	*p* value
Age (months), mean ± SD	85.00 ± 12.86	78.30 ± 22.05	79.52 ± 15.08	0.374
Gender^a^				
Female Male	7 (30.4%) 16 (69.6%)	6 (26.1%) 17 (73.9%)	8 (34.8%) 15 (65.2%)	0.814
IQ, median (IQR)^b^				
Full scale IQ Nonverbal IQ Verbal IQ	103 (92.5–106) 110 (97–112) 99 (92–104.5)	100 (95.5–110.5) 110 (99–110) 100 (90–103)	110 (108–112) 110 (108–111) 110 (106–111)	0.001^*^ 0.370 < 0.001^*^
Fluid reasoning, Mean ± SD	105.87 ± 10.75	103.91 ± 9.63	108.35 ± 6.46	0.263
Knowledge, median (IQR)^b^	103 (97.5–105)	107 (105–111)	109 (103–110)	0.003^*^
Quantitative reasoning, median (IQR)^b^	105 (90.5–108)	100 (93.5–108)	108 (104.5–111)	0.004^*^
Visual spatial reasoning, mean ± SD	100.48 ± 9.36	99.83 ± 9.28	106.22 ± 4.99	0.018^*^
Working memory, Mean ± SD	93.78 ± 7.69	89.70 ± 8.99	108.04 ± 6.23	< 0.001^*^

*Note:* One‐way ANOVA test was used.

Abbreviations: IQ, intelligence quotient; IQR, interquartile range; SD, standard deviation.

^a^ Chi square test.

^b^ Kruskal‐Wallis test was used.

^*^ *p* value ≤ 0.05 is considered statistically significant.

### 3.2 Cognitive Assessment Across the Groups

The results revealed significant differences in Full Scale IQ, Verbal IQ, Knowledge, Quantitative Reasoning, Visual‐Spatial Reasoning, and Working Memory, with the Control group consistently performing better than the Stuttering and Poor Speech groups (*p*‐values ranging from < 0.001 to 0.018), whereas nonverbal IQ and fluid reasoning showed no significant differences (Figure 1). Table 2 shows that pairwise comparisons indicate significant differences in cognitive performance between the groups. Both Group 1 (Stuttering) and Group 2 (Poor Speech) scored lower than the controls in Full Scale IQ (*p* = 0.003 and 0.006, respectively) and Verbal IQ (*p* = 0.0003 and 0.0002, respectively), while Knowledge differed significantly between Group 1 and Group 2 (*p* = 0.0029) and between Group 1 and the controls (*p* = 0.029). Working Memory showed the largest deficits in both Group 1 and Group 2 compared to controls (both *p* < 0.001), and Visual‐Spatial Reasoning was significantly lower in Group 1 compared to controls (*p* = 0.014). Overall, these results indicate that children with stuttering or speech impairments exhibit deficits in specific cognitive domains, particularly in verbal comprehension and working memory, compared to typically developing peers (Figure 2).

**FIGURE 1:**
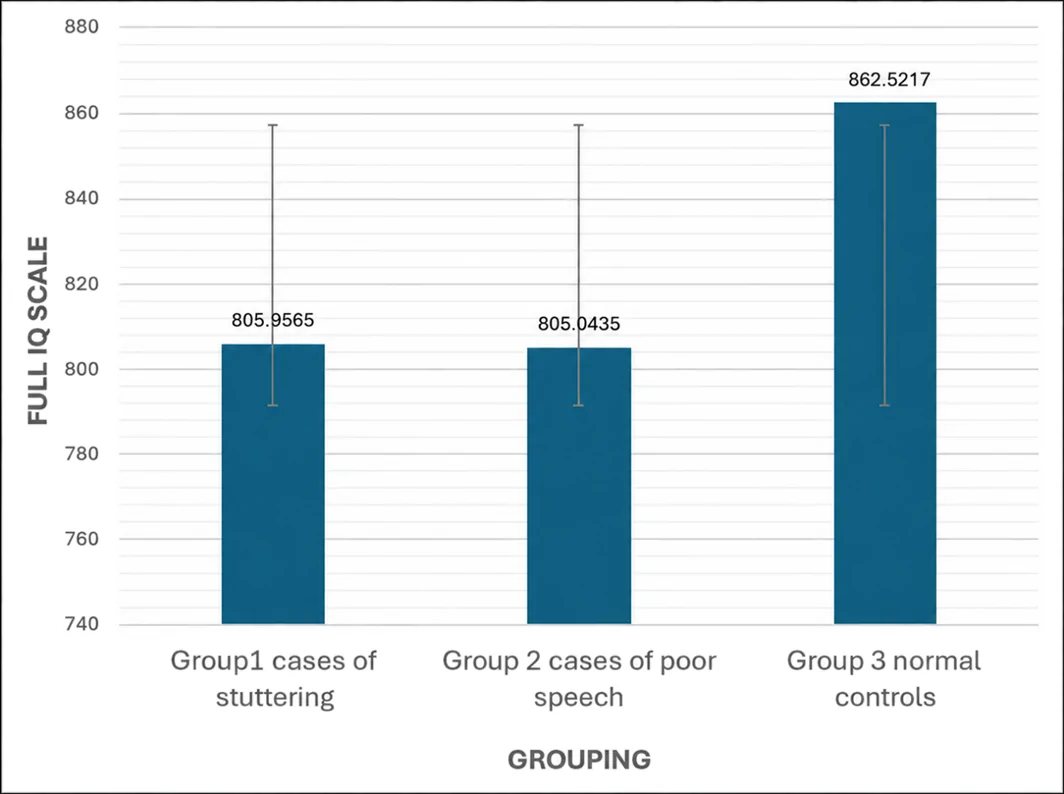
Comparison of full IQ scale among children with stuttering (group 1), poor speech (group 2), and normal controls (group 3).

**TABLE 2:** Post‐hoc comparisons of variables that showed statistical significance.

Variable	Comparison	*p* value
Full scale IQ	Group 1 versus Controls	0.003
	Group 2 versus Controls	0.006
Verbal IQ	Group 1 versus Controls	0.0003
	Group 2 versus Controls	0.0002
Knowledge	Group 1 versus Group 2	0.0029
	Group 1 versus Controls	0.029
Working memory	Group 1 versus Controls	< 0.001^*^
	Group 2 versus Controls	< 0.001^*^
Visual spatial reasoning	Group 1 versus Controls	0.014

^*^ Means difference is statistically significant

**FIGURE 2:**
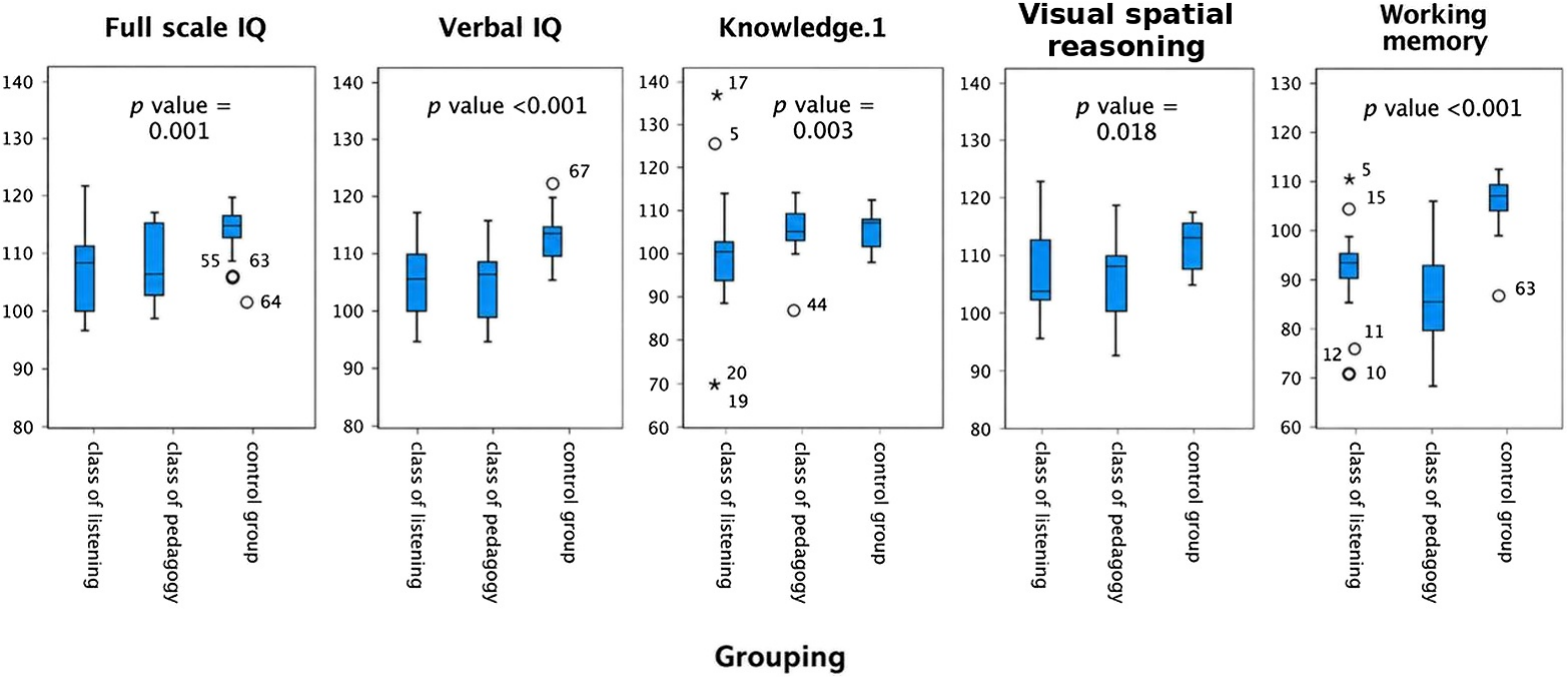
The boxplot for the association between cases of stuttering, poor speech, and controls. (The box represents the interquartile range (Q3–Q1), containing the middle 50% of the data, while the line inside the box indicates the median. The whiskers extend to the lowest and highest values excluding outliers, which are shown as individual points beyond the whiskers).

## 4 Discussion

Children with stuttering and delayed language development exhibited lower overall IQ scores, verbal IQ scores, and deficits across several cognitive domains, including working memory, reasoning, and spatial abilities, compared to typically developing controls. These differences were statistically significant, suggesting that both language delays and fluency disorders are associated with certain domains of cognitive deficits. The present findings revealed significantly lower performance in total IQ scores among children with language impairments and poor speech intelligibility, as well as among those with stuttering, compared to their peers. Although it has often been reported that children with language or fluency disorders have normal cognitive abilities, and that their IQ scores are usually within the average range [15, 29], two studies have reported lower total IQ scores among children who stutter compared to fluent controls [15, 22]. In children with language impairments, a study by Botting [30] found a significantly lower IQ performance by the time they become 8 years. More recent research [31] indicates that while overall intelligence may initially appear similar to typical peers, a noticeable gap in executive function skills appears around the age of six, with children with language impairments performing lower on IQ assessments.

A significant difference was also found in verbal IQ, while nonverbal IQ did not differ significantly between groups. This agrees with the findings of Sami et al. [22], who reported lower verbal performance in children who stutter compared to fluent peers, while their nonverbal scores did not differ significantly from fluent controls. Children with language impairments frequently demonstrate a discrepancy between nonverbal and verbal abilities, with verbal performance being the weaker side [14, 32]. Nonverbal intelligence primarily reflects problem‐solving and reasoning skills without the need to use verbal or linguistic abilities [33], which allows for demonstration of cognitive skills through visual stimuli and gestural responses rather than providing verbal explanations [34]. It has been suggested that children with stuttering or language impairments compensate for the difficulties in providing verbal explanations (either due to language delay or dysfluent speech) by relying more on nonverbal strategies using gestures, pointing, and visual sorting [35]. The present study also found that children with language impairments and stuttering scored significantly lower on the Knowledge subscale, a domain representing crystallized intelligence. This finding further proves deficits in verbal intelligence as this component of intelligence reflects culturally acquired material through education. It is considered a verbal component of intelligence, and it involves retrieving and verbally expressing learned information [36]. Children who stutter may struggle to retrieve and express this knowledge, while holding control of their speech flow [37], whereas those with language impairments often have limited semantic knowledge, poorer vocabulary, and word‐finding difficulties that result in poor verbal expression [38]. Lower performance in visual–spatial processing and working memory was also observed. Visual spatial reasoning depends on problem solving and executive functions such as cognitive flexibility and inhibitory control. Multiple studies have reported executive function deficits in children who stutter [39, 40, 41], and in children with language impairments or multiple phonological errors [24, 42, 43]. Executive functions—particularly inhibition and cognitive flexibility—allow children to shift attention, adapt responses, and suppress irrelevant stimuli [16].

Children with speech and language deficits have problems retrieving and controlling different speech motor acts to control fluency, or struggle in processing and retrieving information in case of language impairment, resulting in defective problem‐solving skills [44]. Working memory stood out as the most significantly affected cognitive domain in both the stuttering and language‐impaired groups. Working memory is one of the main components of executive functions, serving as a limited storage system, whose function is retrieval of information, adapting, and manipulating stored information to produce an appropriate response [45]. Working memory not only depends on recalling information but also on adapting and manipulating this information according to its relevance to the desired stimulus response [46]. Deficits of working memory skills, such as reduced capacity and slower processing, lead to difficulties in retrieval of verbal knowledge, phonological representations of syllables and words, and formulation of long sentences using proper syntactic rules in children with language impairment [24]. Working memory was found lower in children with stuttering and dysfluencies [41, 47]. Poor working memory skills interfere with language comprehension, shifting, adapting, and sustaining attention between cognitive, verbal, and motor speech acts [22]. The co‐occurrence of stuttering and language impairment has been reported in several studies [48], and both conditions have been associated with limited capacity of working memory, leading to slower processing of verbal information, delayed verbal retrieval, and reduced speech fluency [49, 50].

One limitation acknowledged in this study is the small sample size of participants. A larger sample is recommended in future research, to allow for more generalization of the results, as well as testing pre‐ and post‐cognitive therapies and their effects on stuttering and language development. Cognitive therapy outcomes should be examined in follow up research to highlight the conclusions about cognitive therapy in speech and language practice. Though this study focused primarily on standardized tests to ensure validity and objectivity, future research that conducts measures of the complex factors on cognitive and communicative development such as home and school environments, should be considered in future projects, provided that these variables should be assessed via valid and comprehensive questionnaires.

## 5 Conclusion

Children with communication disorders, whether delayed in language development or with speech dysfluencies, demonstrate specific weaknesses in verbal cognitive skills, knowledge, and working memory. Recognizing these subtle cognitive deficits is essential for understanding the mechanisms underlying language and fluency disorders. The findings highlight the importance of integrating cognitive enhancement strategies into traditional speech and language therapy programs. Addressing working memory and verbal reasoning may improve therapeutic outcomes and language fluency. Furthermore, these cognitive aspects are often overlooked in intervention planning, as children typically show average IQ scores despite underlying cognitive inefficiencies. Longitudinal studies are recommended to monitor the impact of cognitive‐based interventions on speech and language improvement over time, providing evidence for more comprehensive and targeted treatment approaches.

## Author Contributions

**Ghada Tarek Orabi:** research idea and management, assessment of language and cognitive profiles of participants, data collection and analysis. **Raghda Abdelsalam Nagaty Hanady:** laboratory analysis of participants, and data collection.

## Funding

The authors have nothing to report.

## Ethics Statement

The study protocol was approved by the Medical Research Ethics Committee, National Research Center, Cairo, Egypt (Federal Wide Assurance No. FWA00014747; RH DIR: 2,017,103,002; Final Decision: 01,451,223) and “Consent for publication”: Written informed consent was obtained from all caregivers prior to participation.

## Conflicts of Interest

The authors declare no conflicts of interest.

## Data Availability

The data that support the findings of this study are available from the corresponding author upon a reasonable request.
